# Treatment of Relapsed Acute Myeloid Leukemia

**DOI:** 10.1007/s11864-020-00765-5

**Published:** 2020-06-29

**Authors:** Felicitas Thol, Arnold Ganser

**Affiliations:** Department of Hematology, Hemostasis, Oncology and Stem Cell Transplantation, Hannover Medical School, Carl-Neuberg-Str. 1, 30625 Hannover, Germany

**Keywords:** Relapse AML, Salavage chemotherapy, Venetoclax, FLT3 inhibitors, IDH1/IDH2 inhibitors, CPX-351

## Abstract

Relapse is still a common scenario in acute myeloid leukemia (AML) treatment and occurs in 40–50% of younger and the great majority of elderly patients. The prognosis in relapsed AML patients is generally poor but depends largely on the timing of relapse (early versus late) and the possibility of allogeneic hematopoietic stem cell transplantation (HSCT). At the time of relapse, we again perform a mutational screening and cytogenetic analysis in all AML patients as clonal evolution of disease is frequent. Clinical trials should be first priority in all relapsed patients. In fit patients without prior transplant, we aim to perform HSCT after salvage therapy. In AML patients relapsing after HSCT and good performance status, intensive therapy can be considered with subsequent cellular therapy such as donor lymphocyte infusion (DLI) or a second HSCT. However, less than 20% of these patients are alive after 5 years. For those patients that are unfit, the therapeutic aim is to prolong life with acceptable quality of life. Here, hypomethylating agents (HMA), low-dose AraC (LDAC), and solely cytoreductive therapy with hydroxurea are options depending on first-line therapy. For those patients that have not been treated with venetoclax in first line, the combination therapy of venetoclax with demethylating agents achieves encouraging response rates. Venetoclax is currently also studied in combination with intensive salvage therapy. Importantly, for patients with isocitrate dehydrogenase (*IDH*) 1/*2*–mutated AML, ivosidenib, an IDH1 inhibitor, and enasidenib, an IDH2 inhibitor, present well-tolerated options in the setting of refractory or relapsed (r/r) disease even in elderly and heavily pre-treated patients with response rates of 30–40%. Both substances have been approved by the U.S. Food and Drug Administration (FDA) for r/r AML patients with *IDH1/2* mutations (but not yet by the European Medicines Agency (EMA)). For patients with FMS-like tyrosine kinase 3 (*FLT3)* mutations, treatment with the selective FLT3 inhibitor gilteritinib is well tolerated and leads to improved outcome compared with standard salvage therapy. The approval has been granted by the FDA and the EMA. Generally, we would recommend targeted therapy for *IDH1/2-* and *FLT3*-mutated AML if available. In order to improve outcome in relapsed AML, it will be important to intelligently combine novel substances with each other as well as chemotherapy in prospective clinical trials. The development of therapies with bispecific antibodies or chimeric antigen receptor T cells (CAR-T) are still in early development.

## Introduction

Despite improvements in acute myeloid leukemia (AML) therapy, relapse is still the most challenging aspect in AML. While 10–40% of younger AML patients are primarily refractory to AML induction therapy, the number is considerably higher for patients above 60 years (40–60%) [1]. The great majority of fit patients will undergo hematopoietic stem cell transplantation (HSCT) after achieving a complete remission (CR). However, 40% of these patients relapse after HSCT [2]. Thus, refractory or relapsed (r/r) AML is a very common scenario in AML. At the time of relapse, a thorough diagnostic work-up is essential. Besides cytology and morphology, we would also recommend performing a cytogenetic analysis as well as mutational screening in all relapsed patients. The need for this analysis at time of relapse is based on the high likelihood of clonal evolution of AML. With the advancement of sequencing technologies, we have learned that the mutational pattern at the time of relapse is frequently discrepant from that at diagnosis. Mutations are frequently gained or lost during the course of the disease. Furthermore, the variant allelic frequency (VAF) of gene mutations is likely to change in the course of the disease, e.g., with smaller clones becoming more dominant as well as dominant clones decreasing in size. The cytogenetic and molecular results help us to further risk stratify patients in relapse. Furthermore, some mutations like *FLT3*, *IDH1*, and *IDH2* offer novel therapeutic options. Due to next-generation sequencing (NGS) being widely available, mutational screening is frequently done in a targeted panel approach covering the most frequently mutated genes in AML. If NGS is not available, we would at least recommend sequencing the “targetable” mutations (e.g., *FLT3*, *IDH1*, *IDH2*) as well as other prognostic mutations (e.g., *NPM1*, *double mutated CEBPA*, *TP53*, *RUNX1*, *ASXL1*) [3••]. Established prognostic relapse scores have integrated molecular markers in the scoring systems such as FLT3-ITD in the GOELAMS score [4]. We recommend human leukocyte antigen (HLA) typing for all patients who are fit for allogeneic stem cell transplantation at the time of diagnosis. However, if this has not been done at that time point, HLA typing and donor search should be initiated as early as possible when relapse occurs. Due to toxicities occurring in the course of the disease and during prior treatment, a through clinical evaluation of patients at the time of relapse is as important as it is at the time of diagnosis and may lead the direction for treatment planning and HSCT. Important questions at the time of relapse include whether the patient can be intensively treated with the aim of subsequent HSCT or other cellular therapies and how likely it is that the patient responds to available relapse therapies.

## Treatment

### Standard treatment for relapse in fit patients with or without prior allogeneic stem cell transplantation

AML patients relapsing after chemotherapy still have a curative treatment approach if they are fit enough to undergo HSCT [5]. Salvage therapy has the aim to induce a cytological remission before HSCT and serves as the “bridge to transplantation”. Clinical trials are our first choice for all eligible patients. There is no standard regimen for salvage therapy and different regimens have shown similar results [5]. However, important components of salvage therapy include an anthracyclin and high-dose cytarabine. Commonly, a purine analog (e.g., fludarabine or cladribine) or etoposide, a topoisomerase II inhibitor, is added to this regimen [5]. A widely used regimen is the FLAG-IDA protocol that includes fludarabine, high-dose cytarabine, idarubicin, and granulocyte colony-stimulating factor (G-CSF). With this regimen, 50% of r/r patients achieve a CR [6]. The FLAG-IDA regimen also presents a frequently used control arm for newer drugs or regimens in clinical trials for r/r AML. The salvage therapy–induced remission is usually only shortly sustained and performing HSCT in a timely manner is critical. For patients relapsing after HSCT, the outlook is even more dismal [7]. An analysis of the Center for International Blood and Marrow Transplant Research (CIBMTR) showed that of 1788 AML patients relapsing after allografts, only 23% of patients were alive at 1 year after relapse [8]. Patients relapsing early after HSCT (< 6 months) have a significantly poorer outcome compared with patients with a late relapse [7, 8]. Especially in the latter group, salvage therapy followed by a repeated HSCT or other cellular therapies such as DLIs can be discussed. There is currently no evidence that a change of donor for second HSCT is of advantage. A European Society for Blood and Marrow Transplantation (EBMT)–based registry study of 418 AML patients relapsing after first HSCT compared outcome of patients receiving a second HSCT compared with DLI [9]. Overall survival (OS) between the two groups was not significantly different with the 2-year OS being 26% and 25% and the 5-year OS being 19% versus 15% respectively [9]. Importantly, this study underscored that outcome of patients with early relapse after HSCT is dismal with 9–11% 2-year OS and 4–9% 5-year OS [9]. Because of these poor results, novel therapies are urgently needed. For specific molecular groups (*FLT3-* or *IDH1/2*-mutated patients), treatment with targeted therapies need to be integrated into therapeutic decision making (see below).

### Relapse in unfit patients after non-intensive therapy

Nearly all elderly patients become refractory or relapse after non-intensive therapy. While HMA treatment such as azacitidine has improved clinical outcome of this patient population, only few patients can be cured with these regimens [10]. While two new substances (venetoclax with HMA or LDAC, glasdegib with LDAC) have been recently approved by the FDA for newly diagnosed elderly AML patients, we still have to learn about the long-term results that can be achieved with these novel combination therapies. Whenever possible, also unfit r/r AML patients should be included in a clinical trial. Approved therapeutic options are very limited in this situation so that cytoreduction with hydroxyurea or LDAC and symptomatic therapy with transfusions and anti-infective therapy might be the only possible therapeutic measures.

## Novel substances—non-targeted

### Venetoclax

Venetoclax is a highly selective, oral small-molecule B cell leukemia/lymphoma-2 (BCL2) inhibitor. Members of the BCL protein family regulate apoptosis in a pro- or anti-apoptotic manner. BCL2 shows anti-apoptotic activity, is highly expressed in leukemia stem cells, and maintains myeloblast survival [11, 12]. Thus, it is not surprising that BCL2 inhibition is a promising target in AML. In a single-arm phase II study, 32 patients with high-risk r/r AML or unfit for intensive chemotherapy received 800 mg venetoclax monotherapy. In this study, monotherapy achieved response rates according to International Working Group (IWG) criteria in 19% of patients while additional 19% of patients showed some antileukemic response that did not fulfill IWG criteria [13]. Similarly, in a very small case serious of seven patients with secondary AML (sAML) who were not eligible for intensive chemotherapy and were refractory to HMA treatment, 2 patients achieved a CR with venetoclax monotherapy that was durable with a PFS of 505 days and 352 days [14]. Both studies indicate that venetoclax monotherapy shows some activity in the r/r AML setting. Nevertheless, these response rates are still unsatisfactory in AML as compared with the impressive results in chronic lymphocytic leukemia. Therefore, the combination therapy of venetoclax is more appealing. Very promising data in first-line treatment of elderly AML patients treated with HMA and venetoclax combination have been reported [15]. In this study, a CR and with incomplete hematological recovery (CRi) was obtained in 67% of patients (91% in *NPM1*-mutated patients). Importantly, favorable remission rates were also observed in prognostically unfavorable subgroups (high-risk cytogenetics, sAML, and *TP53* mutations). The combination of LDAC and venetoclax achieved CR/CRi in 54% of elderly previously untreated AML patients [16]. Here, lower CR/CRi rates were seen in patients with *TP53* or *FLT3* mutations (30% and 44% respectively). These encouraging results led to the approval of venetoclax by the FDA in November 2018 in combination with azacitidine or decitabine or LDAC for the treatment of newly diagnosed AML in adults who are age 75 years or older, or who have comorbidities that preclude use of intensive induction chemotherapy (no approval by EMA yet). Thus, the question comes up how venetoclax combination therapy works in r/r AML patients. In a multicenter historical study, 23 AML patients who were refractory to HMA treatment or relapsed after HMA therapy were treated with a combination of venetoclax and HMA. Forty-three percent achieved a CR or CRi in this cohort while OS was 74% at 6 months [17]. Similar results were seen in 33 r/r AML patients treated with HMA plus venetoclax outside a clinical trial. The overall response rate here was 64% [18]. Thus, combination therapy consisting of HMA and venetoclax is promising in patients with r/r AML. The combination therapy of venetoclax with intensive therapy is currently under investigation. In our observational study, we treated 13 r/r AML patients with FLAG-IDA in combination with venetoclax (FLA-V-IDA; venetoclax given on days 1–7) and compared the results retrospectively with 81 r/r AML patients treated with FLAG-IDA alone [19]. Overall, the venetoclax combination therapy was well tolerated with no excess hematological toxicity. The ORR rate was 69% (FLA-V-IDA) versus 47% (FLAG-IDA). In a similar phase 1B study, r/r AML patients received FLAG-IDA with either venetoclax 200 mg on days 1–21 or subsequently on days 1–14 due to observed infectious complications. For those patients not proceeding to HSCT, venetoclax monotherapy was given as maintenance therapy. Out of 11 evaluable patients, 8 patients (73%) achieved a CR or CRi [20]. Both studies are early but promising. Larger prospective and randomized trials are urgently required to evaluate venetoclax in combination with intensive chemotherapy for fit r/r AML patients. In summary, venetoclax especially in combination therapy is promising in r/r AML.

### CPX-351

CPX-351 is a liposomal formulation of cytarabine and daunorubicin mixed in a 5:1 M ratio. The liposomal drug delivery allows prolonged duration of higher plasma concentrations as well as enrichment of the substances in the bone marrow. CPX-351 was first studied in r/r AML patients and high-risk MDS patients in a dose-finding trial. Of 43 r/r AML patients, 9 patients showed a CR and 1 patient a CRi [21]. In a subsequent randomized phase II trial, CPX-351 was compared with standard salvage chemotherapy in r/r AML patients [22]. In the whole cohort, there was no statistically significant 1-year survival improvement observed. However, in the subgroup of patients with poor risk features patients, the CPX-351 arm showed higher response rates (CR and CRi) (39.3% versus 27.6%) with a statistically significant prolongation of OS (6.6% versus 4.2%) and EFS (2 versus 1.2 months). The observed benefit in r/r AML patients with poor risk features led to a randomized phase III trial in elderly patients with newly diagnosed high-risk AML that included therapy-related AML (t-AML), sAML after MDS or chronic myelomonocytic leukemia (CMML), or de novo AML with MDS-related cytogenetic changes comparing CPX-351 with standard induction therapy [23]. Here, CPX-351 significantly increased median OS (9.56 versus 5.95 months) and overall remission rates (CR and CRi) (47.7% versus 33.3%). The toxicity profile was similar to that seen with standard chemotherapy with the exception of prolonged neutropenia and thrombocytopenia in the investigational arm. These trial results resulted in CPX-351 being approved by the FDA in 2017 and the EMA in 2018 for the treatment of adults with newly diagnosed therapy-related acute myeloid leukemia (t-AML) or acute myeloid leukemia (AML) with myelodysplasia-related changes (AML-MRC). Therefore, despite initially being studied in the r/r AML setting, it is not approved for relapsed AML patients per se. However, it should be considered for patients that meet the AML-MRC criteria and have failed prior HMA therapy for advanced MDS.

### Other non-targeted therapies

Other therapies have emerged in first-line therapy of AML. Glasdegib, a small molecular inhibitor of the sonic hedgehog pathway, has been approved by the FDA for newly diagnosed elderly AML patients in combination with LDAC therapy. The approval was based on phase II study with elderly AML or high-risk MDS patients being randomized to the combination therapy of glasdegib and LDAC (88 patients) versus LDAC alone (44 patients). The median OS was 8.8 months with combination therapy and 4.9 months with LDAC alone [24]. Whether glasdegib also works in the r/r AML setting is currently still unknown.

## Novel substances—targeted

### FLT3 inhibition

*FLT3*-ITD and *FLT3*-TKD mutations are common mutations in AML with frequencies of 20–25% and 6–10%, respectively [25, 26]. Importantly, they are unstable mutations that are frequently lost or gained at the time of relapse. *FLT3*-ITD specifically is a strong adverse prognostic marker at the time of relapse and only a minority of *FLT3*-ITD-positive patients can be cured at the time of relapse despite aggressive salvage therapy [27]. Due to the strong independent adverse effect, *FLT3*-ITD mutation status has been integrated into the prognostic GOELAMS score [4]. Thus, targeting FLT3 with the aim of overcoming the adverse prognostic effect is attractive. Several tyrosinkinase inhibitors have been developed with activity against FLT3 [28]. The first-generation TKIs (e.g., sorafenib, midostaurin) are multitarget inhibitors that function relatively unselectively against FLT3. In contrast, the next-generation TKIs (gilteritinib, quizartinib) have a stronger selectivity for FLT3 [28]. Due to the positive results in the RATIFY trial [29], midostaurin has been approved for *FLT3*-mutated AML in first-line therapy in combination with intensive chemotherapy by the FDA and EMA. It is now standard treatment for newly diagnosed FLT3-mutated AML patients in combination with intensive therapy [29]. However, midostaurin and sorafenib have little activity as monotherapy in relapsed patients [30, 31]. However, next-generation TKIs have shown antileukemic single-agent activity in early clinical trials [28]. Gilteritinib is an oral type 1 FLT inhibitor that was first investigated as monotherapy in 252 r/r AML patients in a phase Ib/II trial [32]. Patients with *FLT3* mutations had the best response with an ORR of 49% compared with 12% in *FLT3* wild-type patients. These promising results in *FLT3*-mutated AML have led the way for a large randomized phase III trial in r/r *FLT3*-mutated AML. Here, monotherapy with gilteritinib was compared with chemotherapy (ADMIRAL trial) [33••]. Chemotherapy in the control arm was performed according to the investigators choice with MEC (mitoxantrone, etoposide, and cytarabine), FLAG-IDA, LDAC, or azacitidine. Of the originally 371 patients enrolled in the trial, 247 patients received gilteritinib and 124 patients standard chemotherapy. The CR rate was 21.1% with gilterinib compared with 10.5 in the comparison arm. Median OS was significantly better in the gilteritinib compared with standard therapy arm (9.3 versus 5.6 months) and 1-year OS was 37.1% versus 16.7%, respectively. The EFS was 2.8 months in the gilteritinib arm and 0.7 months in the control arm. The best results were obtained for patients proceeding to transplant and continuing gilteritinib treatment thereafter. Importantly, subgroup analysis of the trial showed that a response to gilteritinib was also seen in patients with prior use of FLT3 inhibitors while patients with unfavorable cytogenetics did not seem to benefit. The results of the ADMIRAL trial were the basis for the approval of gilterinib in r/r *FLT3*-mutated AML by the FDA. We would recommend gilteritinib for patients with *FLT3*-mutated r/r AML if accessible. Quizartinib is also a next-generation TKI that is relatively selective for FLT3. It was tested in a dose-escalation phase I trial in r/r *FLT3*-ITD-positive AML patients [34]. Here, monotherapy with the substance yielded a CR of 37.5%. In a phase III randomized trial, quizartinib monotherapy was compared with salvage chemotherapy in r/r *FLT3*-ITD-positive AML [35]. Salvage chemotherapy had to be preselected and included LDAC, FLAG-IDA, or MEC. OS was significantly longer in the investigational arm versus standard arm (6.2 versus 4.7 months) with manageable toxicities. Despite the positive phase III results, the FDA and EMA have not granted approval of the substance in r/r *FLT3*-ITD-positive AML patients. Crenolanib, another next-generation TKI with promising results in a phase II trial, is currently also studied in a phase III trial for r/r FLT3-mutated AML (NCT03250338). In summary, next-generation TKIs with specific inhibitory effects for mutated FLT3 are promising in r/r AML. While midostaurin has received approval for FLT3-mutated AML in first line, gilteritinib is now approved for r/r FLT3-mutated AML by the FDA and EMA. It will be interesting to await the results of combination therapy, e.g., gilteritinib with venetoclax, in r/r FLT3-mutated AML (NCT03625505).

### IDH1 and IDH2 inhibitions

*IDH1* and *IDH2* encode for enzymes that catalyze the conversion of isocitrate to a-ketoglutarate in the cytosol and peroxisomes (*IDH1*) and mitochondria (*IDH2*). *IDH1* and *IDH2* mutations are recurrent mutations in AML with a frequency of 7–14% and 8–15%, respectively [26, 36, 37]. A key pathophysiological consequence of mutant *IDH1* and *IDH2* is the production of the oncometablite 2-hydroxgluterate (2-HG) [38]. While *IDH1/2* mutations do not have major prognostic relevance [19, 20], they have gained clinical significance due to the development of targeted therapies. Oral inhibitors have been introduced that inhibit the mutant form of the enzyme and block the production of 2-HG.

Ivosidenib is an oral, targeted, small-molecule inhibitor of mutant IDH1. In a phase I dose-escalation and expansion trial, 258 AML patients with *IDH1* mutations have been treated with ivosidenib [39••]. In this cohort, 179 patients had r/r AML. Of these r/r patients, monotherapy with ivosidenib yielded a CR rate of 21.8%. The median time to CR was 2.8 months and the median duration of CR was 9.3 months. An additional 11.7% of patients achieved a CRi or a CRp (CR with partial platelet recovery) and 38.5% of patients had stable disease. Patients with response to ivosidenib had fewer infections and episodes of febrile neutropenia. No common mutational pattern predicting response or resistance to therapy was identified. Importantly, this therapy was overall well tolerated and allows outpatient oral therapy. Common side effects included prolongation of the QT interval in 7.8% of patients and hematological toxicities in 1.7–3.4% of patients. A special side effect to IDH inhibition is the IDH differentiation syndrome (DS) which occurred in 3.9% of patients in this study [39••]. This clinical scenario is a potentially fatal complication of IDH inhibitor therapy and similar to that seen in acute promyelocytic leukemia during treatment with all-trans retinoic acid and/or arsenic trioxide. Symptoms include dyspnea, unexplained fever, pulmonary infiltrates, hypoxia, and pleural effusions. These patients can show leukocytosis with signs of maturation; however, it is not mandatory. It is critical to identify IDH-DS early and treat it with corticosteroids. Similar to ivosidenib for *IDH1-*mutant AML, enasidenib has been developed for *IDH2*-mutant AML. Enasidenib is an allosteric inhibitor that binds to the IDH2 dimer interface and blocks the production of 2-HG of IDH2 mutants. In a phase I/II trial dose-escalation and expansion trial, 239 patients with *IDH2*-mutant advanced myeloid malignancies were treated [40, 41••]. Among this cohort, 176 patients had r/r disease. In patients with r/r AML, enasidenib therapy yielded an overall response rate of 40.3%. The median time to first response was 1.9 months and 87.3% of responding patients showed a first response by cycle 5. One hundred milligrams of enasidenib daily was chosen as the dose for the expansion cohort based on plasma drug concentration, 2-HG inhibition, and clinical activity. With 100 mg enasidenib daily, 38.5% of r/r patients achieved a response and 20.2% of patients a CR. The duration of response ranged from 3.8 to 9.7 months (median 5.6 months). The median OS for r/r was 9.3 months with an estimated 1-year OS of 39%. Interestingly, the drug works independent of the mutational load of *IDH2*. A rise in indirect bilirubin serum level was observed in 35% of patients. However, it was not a sign of intrinsic liver toxicity but instead due to off-target inhibition of the UGT1A1 enzyme. 9.6% of patients developed IDH-DS, with grade 3 or 4 in 6% of patients. The mechanisms underlying the evolving resistance to IDH inhibition after initial response have been studied. It was shown that resistance to evosidenib can emerge through the development of second-site *IDH2* mutations in *trans* that inhibit the binding of the drug [42]. Ivosidenib and enasidenib represent an important progress in the treatment of r/r AML patients with targeted therapy. Both drugs have been approved by the FDA for r/r AML patients with an IDH1 and IDH2 mutation respectively. Current clinical trials concentrate on combination therapies of ivosidenib or enasidenib with azacitidine (NCT03683433) or CPX-351 (NCT03825796) in r/r AML patients as well as with induction chemotherapy in newly diagnosed patients (NCT03839771).

## Future directions

Currently, we have little data about the efficacy and toxicity of combining novel substances with each other or with chemotherapy in r/r AML. It will be important to gain prospective data in randomized trials that study venetoclax with salvage chemotherapy or other targeted therapies (e.g., gilteritinib for *FLT3*-mutated r/r AML or ivosidenib/enasidenib in *IDH1-/IDH2*-mutated r/r AML). Cellular therapies such as CAR-T cells and bispecific antibody are less advanced in AML compared with ALL. CD33- and CD123-directed bispecific antibodies have been developed. These bispecific antibodies are also called dual-affinity retargeted antibodies. AMG 330 is anti-CD33 bispecific T cell engager antibody construct that was studied in r/r AML patients in a phase I trial. It was a dose-escalation study that intended to find the optimal dosing schedule. Of 40 patients, the majority of patients discontinued therapy (*n* = 35) with a median of one cycle being applied. Of the 35 patients who discontinued therapy, 27 patients (77%) discontinued due to disease progression. A total of 73% of patients experienced serious adverse events with cytokine release syndrome being very common (28% of patients) [43]. Similar dose-finding studies are ongoing with flotetuzumab, a CD123xCD3 dual-affinity retargeted antibody. CD123 is also target of a donor-derived anti-CD123-CAR-T cell that is currently tested in relapsed AML after allo-HSCT (NCT03114670). However, finding the ideal target remains challenging both for dual-affinity retargeted antibodies as well as for CAR-T cell therapy.

## Conclusion

R/R AML remains a common clinical scenario with poor outcome. HSCT is necessary for long-term remission and survival. Due to the limited treatment options in relapsed patients, clinical trials are the first choice of therapy. For *FLT3-* and *IDH1-/IDH2*-mutated AML patients, targeted therapies have shown results superior to standard therapy. The FDA has approved gilteritinib for *FLT3-*mutated AML as well as ivosidenib/enasidenib for *IDH1-/IDH2-*mutated r/r AML patients. Monotherapy with venetoclax, a bcl-2 inhibitor, has moderate efficacy in r/r AML. However, early results in combination with intensive chemotherapy or HMA are very encouraging. Trials combining novel substances with each are necessary. The development of cellular therapies such as CAR-T cells or bispecific T cell engager antibody constructs is very demanding in AML as it is difficult to identify the ideal target structure on AML blasts.
